# Association Between Visual Acuity and Cycloplegic Refractive Error in 3- to <10-Year-Old Children

**DOI:** 10.1007/s44402-026-00051-1

**Published:** 2026-03-11

**Authors:** Jennifer X. Haensel, Angela M. Chen, Aparna Raghuram, Vivian M. Manh, Susan A. Cotter, Lisa A. Jones-Jordan, Silvia Han, Ingryd Lorenzana, Kristine Huang, Reena Patel, Amy A. Lytle, Dashaini Retnasothie, Tawna L. Roberts

**Affiliations:** 1 Spencer Center for Vision Research, Byers Eye Institute at Stanford University, Palo Alto, California USA; 2 Southern California College of Optometry at Marshall B. Ketchum University, Fullerton, California USA; 3 Boston Children’s Hospital, Harvard Medical School, Boston, Massachusetts USA; 4 Seattle Children’s Hospital, Seattle, Washington USA; 5 The Ohio State University College of Optometry, Columbus, Ohio USA; 6 Advanced Vision Center, Schaumburg, llinois USA; 7 Snowy Range Vision Center, Laramie, Wyoming USA

**Keywords:** Cycloplegia, Refractive error, Visual acuity, Visual acuity testing

## Abstract

**Purpose:**

Distance visual acuity (VA) has been associated with refractive error in older children, but less is known about children <6 years of age and those without a history of refractive correction. This study examined the utility of VA testing and its relationship to refractive error in children aged 3 to <10 years without a history of refractive correction.

**Methods:**

Unaided monocular distance VA testing was performed at 3 m (age 3–6 years: ATS-HOTV chart; 7–<10 years: E-ETDRS chart) and near VA at 40 cm (ATS-4 Near VA). Cycloplegic autorefraction was used to categorise participants as myopic (sphere ≤ −0.75 dioptres (D)), hyperopic (sphere ≥2 D), astigmatic (cylinder ≥1.50 D) and emmetropic (< 0.75 D myopia and <2 D hyperopia). Receiver operating characteristic curves assessed the utility of VA testing in classifying children by refractive error type. Linear regressions examined the predictive value of refractive error magnitude in determining distance and near VA while accounting for age.

**Results:**

Of 358 children, 84 (23.5%) had hyperopia, 30 (8.4%) myopia, 39 (10.9%) astigmatism and 229 (64.0%) emmetropia. Reduced distance VA was associated with myopia (area under the curve (AUC) = 91%, optimal cut-off = 0.15 logMAR) and astigmatism (AUC = 87%, cut-off = 0.25 logMAR), but not hyperopia (AUC = 63%, cut-off = 0.05 logMAR). Near VA showed only mildly higher performance for hyperopia (AUC = 70%, cut-off = 0.15 logMAR). For every 0.36 D increase in myopia, distance VA declined by 0.10 logMAR (*p* < 0.001). Distance and near VA were not predicted by the magnitude of hyperopia (distance: *p* = 0.30; near: *p* = 0.30) or astigmatism (distance: *p* = 0.35; near: *p* = 0.06).

**Conclusion:**

In children 3 to <10 years of age without prior refractive correction, reduced distance VA was associated with myopia and astigmatism, but not hyperopia; an incremental decline in VA with increasing refractive error magnitude was seen only in myopia.

Key Points

- Distance visual acuity was associated with clinically significant myopia and astigmatism, but not hyperopia, in 3- to <10-year-olds without a history of refractive correction.
- Using age-normed visual acuity thresholds did not improve the utility of visual acuity testing to identify hyperopia and astigmatism.
- Reductions in distance visual acuity with increasing refractive error were observed only in cases of myopia, but not hyperopia or astigmatism.

## Introduction

Uncorrected refractive errors in children can result in reduced visual acuity (VA) and negatively impact quality of life, academic and educational outcomes, as well as overall development [1–4]. Timely detection of clinically significant refractive error and subsequent optical correction is especially critical in young children, who are at higher risk of developing vision disorders such as amblyopia or strabismus [5–8]. Distance VA, a primary visual function metric of the eye, is used in both vision screening and clinical settings. It can be administered rapidly with high test–retest reliability in 3- to 7-year-olds [9, 10], with children as young as 3 years of age typically cooperating when age-appropriate optotypes are used [9–11]. In vision screening settings, examiners use VA measures to identify children with possible underlying refractive error and/or ocular disease who would require a referral to an eye care provider for further examination. In clinical settings, eye care providers evaluate the relationship between VA and refractive error to help determine whether reduced VA is due to refractive error or an underlying ocular pathology. Thus, it is important to understand the association between VA and refractive error.

Prior studies on the relationship between VA and refractive error have shown distance VA to be a useful measure in children 6 years and older when identifying uncorrected myopia and astigmatism, but not hyperopia [12–15], except in cases of high hyperopia (e.g., ≥4.50 dioptres (D) in 6- to 12-year-olds) [16]. However, less is known about the relationship between VA and the different types of refractive error in children younger than 6 years of age, who may further present with normally reduced VA levels compared with older children due to lower attentional and cognitive capacity [17, 18]. It is unclear how the use of age-adjusted VA norms in children aged 3–6 years impacts the expected associations between distance VA and refractive error. While distance VA has been shown to be poorly associated with any type of clinically significant refractive error in 3- to 5-year-olds [11], reduced VA has been found to be characteristic of moderate myopia (≥ 3.00 D myopia), high hyperopia (≥ 4.50 D hyperopia) and astigmatism (≥ 2.00 D) in 2- to 7-year-olds [19]. However, associations between VA and different types of refractive error of lower, yet still clinically significant, magnitude remain to be studied. Given the discrepancy in findings in the literature, associations between VA levels and different types of refractive error in younger children remain inadequately understood and warrant further study. In addition, while prior studies typically measured uncorrected VA, their study samples included children with a history of wearing a refractive correction [14, 15]. Studies with adults have demonstrated significant visual adaptations to refractive correction or lenses that impact VA [20] and blur perception [21], suggesting that even just brief periods of refractive wear can impact VA testing. Thus, studies involving children without a history of refractive corrections are needed.

The objective of the current study was to examine the associations between distance VA and refractive error type (i.e., clinically significant myopia, hyperopia and astigmatism) in children aged 3 to <10 years without a history of refractive correction. Near VA was also examined since it has been reported to outperform distance VA in detecting hyperopia in 6- to 12-year-olds [16]. In addition, associations between age-normed VA thresholds and refractive error types were assessed, as well as whether the magnitude of uncorrected refractive error predicted VA.

## Methods

### Participants

Participants were recruited from the local community and during routine eye examinations at the following eight clinical sites as part of a larger study on visual function in children with uncorrected refractive error: Advanced Vision Center, Illinois; Akron Children’s Hospital, Ohio; Boston Children’s Hospital, Massachusetts; Illinois Eye Institute, Illinois; Seattle Children’s Hospital, Washington; Snowy Range Vision Center, Wyoming; Southern California College of Optometry at Marshall B. Ketchum University, California and Stanford Medicine Children’s Health, California. Inclusion criteria were 3 to <10 years of age and no previous or current refractive correction. Exclusion criteria included neurological disease, medications known to affect the oculomotor system and developmental delay that would interfere with testing. The study was conducted in accordance with the Declaration of Helsinki and was approved by the local Institutional Review Board at each individual site. The parent or legal guardian provided written permission for their child to take part in the study, and children aged 7 to <10 years provided written assent.

### Procedures

Unaided VA was assessed monocularly at 3 m using single-surrounded optotypes based on the Amblyopia Treatment Study (ATS)-HOTV [9, 10] (3 to <7 years) or Electronic-Early Treatment of Diabetic Retinopathy Study (E-ETDRS) [22, 23] (7 to <10 years) protocols. Unaided monocular near VA was assessed at 40 cm using the ATS-4 near VA test [24, 25]. Thirty minutes after instilling two drops of 1% cyclopentolate, cycloplegic refraction was measured using the Grand Seiko WAM-5500 autorefractor (Rexxam Co. Ltd., grandseiko.com) in minus-cylinder notation while participants monocularly viewed a star-shaped stimulus (14 cm in diameter) with a centred dot (2 cm in diameter) to hold fixation at 3 m. A minimum of five static measurements were recorded.

### Data Processing

#### Coding of distance/near VA and selection of eye for analysis

ATS-HOTV VA represented the lowest logMAR level at which three of three or three of four letters were identified correctly [9, 10], while E-ETDRS VA was obtained as a letter score [22, 23]. For analysis, VA was expressed as logMAR units to enable pooling of data across age groups. The eye with better distance VA was used for statistical analysis. When the distance VA was equal in both eyes, the right eye was analysed; if available for only one eye, that eye was used. Age-normed VA values were also applied to account for the expected reduction in VA in younger children [18]. Specifically, measurements were re-coded such that the value of 0 corresponded to the typical VA cut-off for each age group: 0.10 logMAR (6/7.5 Snellen equivalent) for ≥7 years of age, 0.20 logMAR (6/9.6) for 5 and 6 years of age, 0.30 logMAR (6/12) for 4 years of age and 0.40 logMAR (6/15) for 3 years of age [26]. VA measurements below or above 0 (i.e., the cut-off in each age group) were re-coded accordingly. For example, a measurement of 0.60 logMAR for a 3-year-old would be re-coded as 0.20 (i.e., 0.20 logMAR above the age-adjusted cut-off) and for a 6-year-old as 0.40 (i.e., 0.40 logMAR above the cut-off). Similar procedures were applied to near VA measurements. Near VA findings were taken from the same eye analysed for the distance measurements.

#### Cycloplegic autorefraction measurements

Measurements of cycloplegic autorefraction were filtered offline to remove spurious readings if the sphere power or cylinder power exceeded the mode by 1.00 D. The middle five readings were then converted to power vectors, averaged to obtain mean spherical equivalent (SE), J_0_ and J_45_ values [26], and back-transformed to sphero-cylindrical notation using minus-cylinder form.

#### Refractive error classification

All refractive error classifications were based on minus-cylinder notation. Participants were considered to have clinically significant *myopia* if the cycloplegic sphere value was ≥0.75 D myopia, *hyperopia* if sphere was ≥2 D hyperopia, *astigmatism* if cylinder was ≥1.50 D [27] and *emmetropia* if they had between <0.75 D myopia and <2 D hyperopia and <1.50 D astigmatism. Since analyses were conducted separately for each refractive error classification, a participant with more than one refractive error, such as having both hyperopia and astigmatism, was counted in all relevant classifications. Participants were classified as *having refractive error* if they were classified as myopic, hyperopic or astigmatic and as *not having refractive error* if they were classified as emmetropic.

### Data Analysis

To assess the utility of distance and near VA in classifying different types of refractive error, receiver operating characteristic (ROC) curves using continuous logMAR values were generated, along with sensitivity, specificity, area under the curve (AUC) metrics and optimal logMAR cut-off points based on the Youden Index [28]. Cut-off points were also mapped to the nearest Snellen equivalent values for clinical interpretability.

Linear regressions were performed to assess the predictive value of refractive error magnitude and age in determining distance or near VA. For each refractive error type—myopia only (without astigmatism), hyperopia only (without astigmatism) and astigmatism-only (without myopia or hyperopia)—a separate linear regression was conducted using sphere power (in the case of myopia and hyperopia) or cylinder power (astigmatism), as well as age as predictor variables and distance or near VA as the outcome variable. Participants with more than one refractive error classification (e.g., both myopia and astigmatism) were removed from this analysis to better isolate the impact of each individual refractive error type on VA.

### Ethics and Consent to Participate Declaration

Prior to participating in the study, participants aged ≥7 years gave their written assent, and their parent or guardian gave written informed consent.

## Results

### Participants

Of 390 participants tested, 32 were excluded from analysis due to missing data for age (*n* = 8), distance VA (*n* = 14), near VA (*n* = 4) or cycloplegic refraction (*n* = 6). This resulted in a final analytic sample of 358 participants with a median age interquartile range of 6 (2) years (3 years: *n* = 9, 2.5%; 4 years: *n* = 52, 14.5%; 5 years: *n* = 98, 27.4%; 6 years: *n* = 59, 16.5%; 7 years: *n* = 55, 15.4%; 8 years: *n* = 44, 12.3%; 9 years: *n* = 41, 11.5%).

### Refractive Error

Of the 358 included participants, 84 were hyperopic (23.5%), 30 myopic (8.4%) and 229 emmetropic (64.0%). Thirty-nine of the 358 children (10.9%) had astigmatism, of whom 22 were hyperopic, 2 were myopic and 15 children had astigmatism without classifications of myopia or hyperopia (see 'Refractive error classification'). In total, 129 of 358 participants (36.0%) met the criteria for having clinically significant refractive error (hyperopia, myopia and/or astigmatism), while 29 (64.0%) did not (emmetropia).

**Fig. 1:**
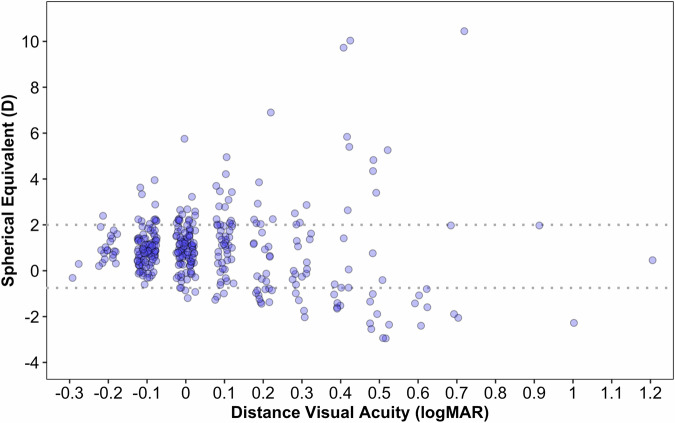
Distribution of spherical equivalent refractive error in dioptres (D) versus logMAR distance visual acuity (VA) measures without age adjustment. The grey dotted lines represent sphere thresholds for hyperopia (2.00 D) and myopia (−0.75 D).

**Fig. 2:**
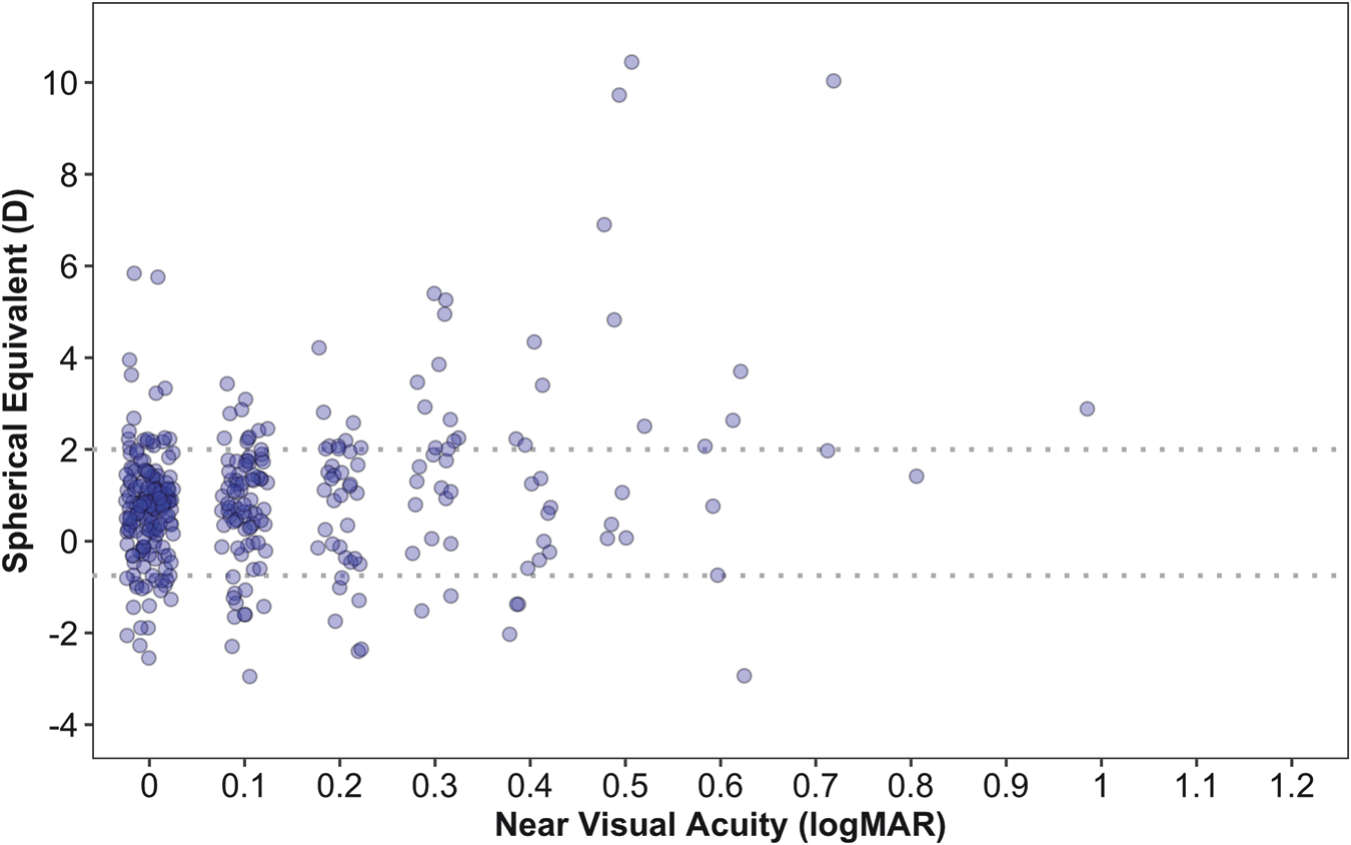
Distribution of spherical equivalent refractive error in dioptres (D) versus logMAR near visual acuity (VA) measures without age adjustment. The grey dotted lines represent sphere thresholds for hyperopia (2.00 D) and myopia (−0.75 D).

For visualisation, Figs. 1 and 2 show the association between VA measures without age adjustment and SE refractive error. Median distance VA was worse in myopia (0.40 logMAR), astigmatism (0.30 logMAR) and all refractive errors combined (0.20 logMAR) than for emmetropia (0.0 logMAR) or hyperopia (0.10 logMAR); however, there was a wide range of VA values (Table 1). Median near VA was worse in astigmatism (0.40 logMAR) and hyperopia (0.20 logMAR) than myopia (0.10 logMAR) or emmetropia (0.0 logMAR; Table 1).

**Table 1:** Medians (IQR) and ranges for age, RE (minus cylinder) and VA.

	Age in years	Sphere (D)	Cylinder (D)	SE (D)	Distance VA (logMAR)	Near VA (logMAR)
All (*n* = 358)	6 (2)	+1.17 (1.45) −2.49 to +11.59	−0.47 (0.60) −4.24 to −0.02	+0.87 (1.50) −2.95 to +10.45	0.0 (0.2) −0.30 to 1.20	0.0 (0.1) 0.0 to 1.30
Emmetropia (*n* = 229)	6 (3)	+0.98 (0.98) −0.73 to +1.99	−0.39 (0.39) −1.39 to −0.02	+0.77 (0.88) −1.14 to +1.90	0.0 (0.1) −0.30 to 1.20	0.0 (0.1) 0.0 to 1.30
Hyperopia (*n* = 84)	5 (1)	+2.74 (1.45) +2.00 to +11.59	−0.70 (1.25) −4.24 to −0.07	+2.23 (1.13) +0.76 to +10.45	0.10 (0.2) −0.20 to 0.90	0.20 (0.3) 0.0 to 1.30
Myopia (*n* = 30)	6.5 (3)	−1.19 (0.79) −2.49 to −0.75	−0.49 (0.51) −2.67 to −0.07	−1.52 (0.84) −2.95 to −0.86	0.40 (0.3) 0.0 to 1.00	0.10 (0.1) 0.0 to 0.60
Astigmatism (*n* = 39)	5 (2)	+2.54 (3.49) −1.60 to +11.59	−2.46 (1.07) −4.24 to −1.54	+1.25 (3.00) −2.94 to +10.45	0.30 (0.2) 0.0 to 0.70	0.40 (0.3) 0.0 to 0.80
All REs (*n* = 129)	5 (2)	+2.26 (2.85) −2.49 to +11.59	−0.77 (1.33) −4.24 to −0.07	+1.98 (3.31) −2.95 to +10.45	0.20 (0.4) −0.20 to 1.00	0.20 (0.3) 0.0 to 1.30

*D* dioptre, *IQR* interquartile range, *RE* refractive error, *SE* spherical equivalent, *VA* visual acuity.

### Utility of Distance VA Testing

**Fig. 3:**
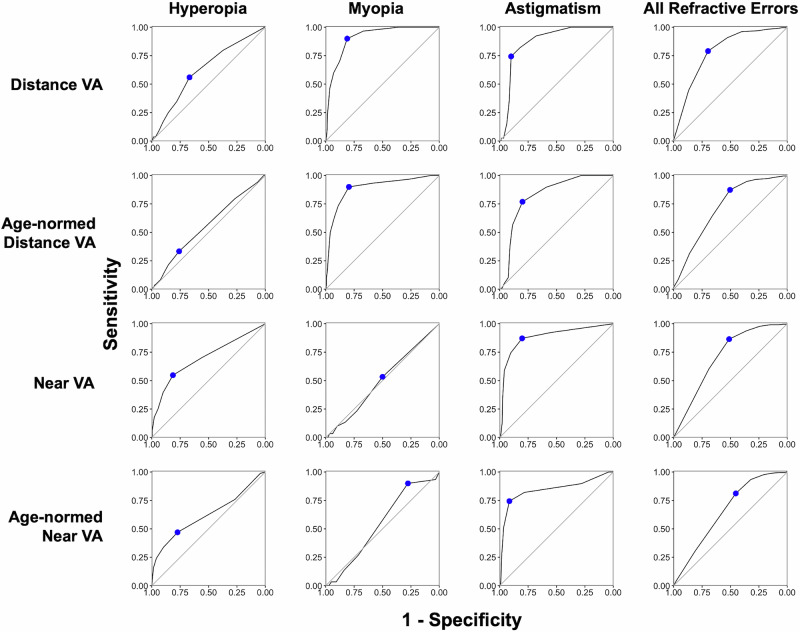
Receiver operating characteristic (ROC) curves for each refractive error classification using distance visual acuity (VA) without age adjustment (top row), age-normed distance VA (second row), near VA without age adjustment (third row) and age-normed near VA (bottom row). The blue dot corresponds to the threshold based on the Youden Index.

For distance VA measures without age adjustment, the highest AUCs were observed for myopia (91.0%) and astigmatism (87.2%; Fig. 3, top row; Table 2). Corresponding sensitivity and specificity values ranged from 74.0% to 90.0% (Table 2), with a VA cut-off of 0.15 logMAR for myopia (corresponding to the closest VA line of 0.20 logMAR or 6/9.6 Snellen equivalent) and 0.25 logMAR for astigmatism (closest line: 6/12 Snellen equivalent). Compared with myopia and astigmatism, the ROC curve for all refractive errors combined showed a slightly lower but moderate AUC (79.0%; sensitivity: 79.0%, specificity: 69.8%), with a cut-off of 0.05 logMAR (closest line: 6/7.5 Snellen equivalent). Distance VA was poorly associated with hyperopia (AUC: 62.6%; sensitivity: 56.0%, specificity: 66.8%), with a cut-off at 0.05 logMAR. AUCs and sensitivity–specificity pairs were lower for age-normed VA measures, compared with unadjusted measures (Fig. 3, second row; Table 3).

**Table 2:** ROC curve results for each RE classification using distance and near VA measures without age adjustment.

Classification	RE	Sensitivity	Specificity	AUC	VA cut-off (logMAR)	Snellen equivalent
Distance VA	Myopia	90.0%	81.1%	91.0%	0.15	6/9.6
	Hyperopia	56.0%	66.8%	62.6%	0.05	6/7.5
	Astigmatism	74.4%	90.0%	87.2%	0.25	6/12
	All RE	79.0%	69.8%	79.0%	0.05	6/7.5
Near VA	Myopia	53.3%	50.0%	50.1%	0.05	6/7.5
	Hyperopia	54.8%	81.4%	70.0%	0.15	6/9.6
	Astigmatism	87.2%	80.3%	88.3%	0.15	6/9.6
	All RE	86.5%	51.2%	71.3%	0.15	6/9.6

*AUC* area under the curve, *RE* refractive error, *ROC* receiver operating characteristic, *VA* visual acuity.

**Table 3:** ROC curve results for each RE classification using age-normed distance and near VA measures.

Classification	RE	Sensitivity	Specificity	AUC	logMAR VA cut-off (Snellen equivalent) by age			
					3 years	4 years	5–6 years	≥7 years
Distance VA (age-normed)	Myopia	90.0%	79.6%	89.0%	0.35 (6/15)	0.25 (6/12)	0.15 (6/9.6)	0.05 (6/7.5)
	Hyperopia	33.3%	75.9%	55.7%	0.35 (6/15)	0.25 (6/12)	0.15 (6/9.6)	0.05 (6/7.5)
	Astigmatism	76.9%	79.9%	82.5%	0.35 (6/15)	0.25 (6/12)	0.15 (6/9.6)	0.05 (6/7.5)
	All RE	87.3%	50.4%	71.8%	0.35 (6/15)	0.25 (6/12)	0.15 (6/9.6)	0.05 (6/7.5)
Near VA (age-normed)	Myopia	90.0%	27.6%	54.3%	0.25 (6/12)	0.15 (6/9.6)	0.05 (6/7.5)	-0.05 (6/6)
	Hyperopia	47.0%	77.3%	62.4%	0.35 (6/15)	0.25 (6/12)	0.15 (6/9.6)	0.05 (6/7.5)
	Astigmatism	74.4%	91.5%	84.3%	0.45 (6/18.9)	0.35 (6/15)	0.25 (6/12)	0.15 (6/9.6)
	All RE	81.1%	45.3%	65.8%	0.35 (6/15)	0.25 (6/12)	0.15 (6/9.6)	0.05 (6/7.5)

*AUC* area under the curve, *RE* refractive error, *ROC* receiver operating characteristic, *VA* visual acuity.

### Utility of Near VA Testing

With chance-level performance (AUC: 50.1%), near VA measures without age adjustment were poorly associated with myopia, which was expected given that the accommodative demand was 2.50 D (40 cm) and the highest level of myopia observed in the present dataset was 2.49 D. Hyperopia showed a higher AUC (70.0%, compared with 62.7% for distance VA) with a 0.15 logMAR cut-off (closest line: 6/9.6 Snellen equivalent; Table 2). The ROC curve metrics for astigmatism (AUC: 88.3%) were similar to distance VA (AUC: 87.2%). For all refractive errors combined, the AUC indicated slightly reduced performance using near (71.3%) compared to distance VA (79.0%), with a cut-off at 0.15 logMAR (closest line: 6/9.6 Snellen equivalent). For age-normed near acuity measures (Table 3; Fig. 3, bottom row), AUCs were reduced compared to unadjusted near acuity measures except for myopia, although performance remained poor (54.3%).

### Effect of Refractive Error and Age on VA

**Fig. 4:**
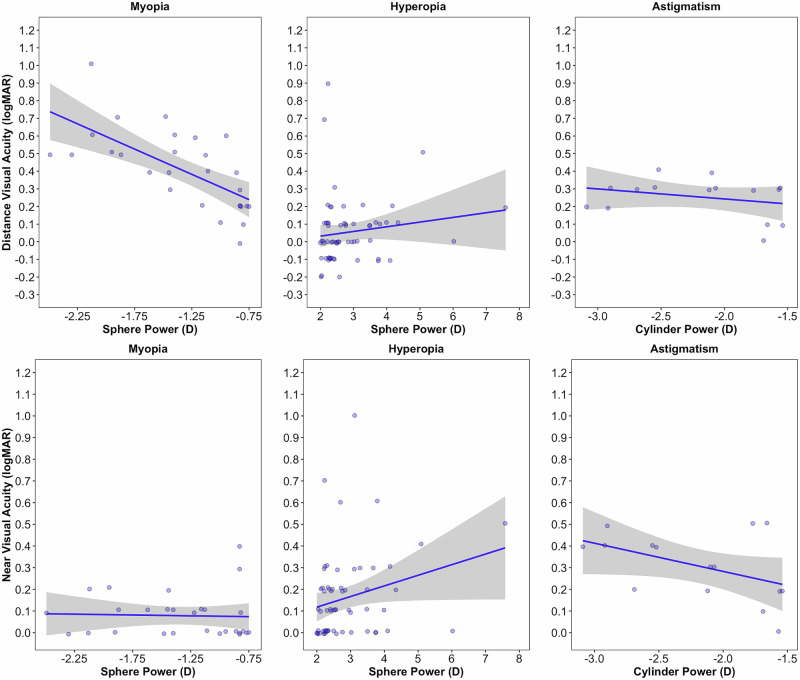
Scatterplots depicting the association between distance (top) and near visual acuity (VA) (bottom), in logMAR, and magnitude of sphere or cylinder power (in D) for myopia (left), hyperopia (middle) and astigmatism (right). The regression line is plotted (in blue) along with the 95% confidence intervals (shaded area). Negligible vertical jitter was applied to avoid overlapping points. D dioptres.

Regression analyses demonstrated that in myopia (*n* = 28), the sphere power significantly predicted distance VA levels (*p* < 0.001), whereas age did not (*p* = 0.14; see Table 4 for full statistical details, including adjusted *R*^2^ values). Distance VA declined by 0.10 logMAR per 0.36 D increase in myopia. However, for hyperopia (*n* = 62) and astigmatism (*n* = 15), neither the magnitude of refractive error (sphere power for hyperopia and cylinder power for astigmatism) nor age predicted distance VA significantly (see also Fig. 4). Unlike for distance VA, sphere power did not predict near VA levels in myopia (*p* = 0.73) or hyperopia (*p* = 0.30). Similarly, cylinder power did not predict near VA in astigmatism (*p* = 0.06). Age was a significant predictor of near VA in myopic participants (*p* < 0.001), with acuity improving by 0.10 logMAR for every 3-year increase in age.

**Table 4:** Statistical values for the linear regressions using sphere power for myopia (*n* = 28) and hyperopia (*n* = 62) and cylinder power for astigmatism (*n* = 15), as well as age to predict distance and near VA levels.

VA test	Model	Adj. *R*^2^	Variable	Estimate	95% CI	*p* value
Distance VA	Myopia	45.9%	Sphere	−0.28	[−0.41, −0.16]	<0.001
			Age	0.03	[−0.01, 0.06]	0.14
	Hyperopia	<0.01%	Sphere	0.02	[−0.02, 0.07]	0.30
			Age	−0.02	[−0.05, 0.01]	0.27
	Astigmatism	<0.00%	Cylinder	−0.06	[−0.18, 0.07]	0.35
			Age	0.002	[−0.03, 0.04]	0.93
Near VA	Myopia	31.5%	Sphere	−0.01	[−0.08, 0.05]	0.73
			Age	−0.03	[−0.05, −0.02]	<0.001
	Hyperopia	4.44%	Sphere	0.03	[−0.03, 0.09]	0.30
			Age	−0.04	[−0.08, <0.01]	0.07
	Astigmatism	15.9%	Cylinder	−0.14	[−0.29, 0.01]	0.06
			Age	−0.02	[−0.06, 0.02]	0.34

*Adj. R*^*2*^ adjusted *R*^2^, *CI* confidence interval, *VA* visual acuity.

## Discussion

Consistent with previous studies of mostly older children, reduced distance VA was associated with myopia and astigmatism, but not hyperopia [12–15], in this sample of 3- to <10-year-old children without a history of refractive correction. Near VA showed improvements in the association with hyperopia, but remained poor, missing half of the cases at the 0.20 logMAR cut-off point. While lower levels of clinically significant hyperopia (e.g., 2.00 D) may not be associated with reduced distance or near VA at this age, clinically significant hyperopia was defined as ≥2.00 D due to the increased risk of developing vision conditions such as esotropia [5, 7, 8].

For distance VA, optimal cut-off points for myopia and astigmatism of 0.15 and 0.25 logMAR, respectively, as identified in the present study are in line with previous investigations reporting cut-off points between 0.20 and 0.30 logMAR for children ≥6 years [12–16, 29], as well as with established vision screening guidelines of 0.30 and 0.20 logMAR for children 4 and ≥5 years, respectively [20]. For hyperopia, a 0.05 logMAR cut-off point was identified, which is considered normal VA. This is consistent with previously reported cut-off points between 0.00 and 0.12 logMAR [12, 14], and further supports that distance VA is poorly associated with hyperopia. In contrast to previous studies reporting cut-off points between 0.14 and 0.22 logMAR for any type of refractive error [12, 14], a 0.05 logMAR cut-off point, coupled with lower AUC, sensitivity and specificity values, was identified. The poorer performance of distance VA in classifying general refractive error in the current study may be due to the higher proportion of hyperopic children in the sample. In contrast, previous studies included substantially more cases of myopia and astigmatism [12, 14], which likely enhanced model performance given the greater effectiveness of VA testing for these refractive errors. Another reason for the lower performance in classifying general refractive error could be due to a poorer diagnostic value of distance VA for younger ages (< 6 years), which has been supported by previous findings showing lower performance in 6- to 7-year-olds compared with 12- to 13-year-olds [12]. While the AUC findings suggest that distance VA performance was slightly worse in children <6 years, the difference was small compared with children ≥6 years of age (data not shown due to duplicity in reporting of results). Differences in refractive error definitions may also explain the discrepancy. For example, O’Donoghue et al. [12] used thresholds of >3.50 D hyperopia and ≥1.00 D myopia, whereas the present study used ≥2.00 D hyperopia and ≥0.75 D myopia. Since higher refractive errors are typically associated with poorer distance VA, studies using higher thresholds are more likely to find stronger associations between refractive error and distance VA. For example, 24 children in the current study had ≥3.50 D hyperopia, which was associated with slightly improved classification performance (AUC: 74.8%, data not shown due to limited sample size) compared with an AUC of 62.6% using the ≥2.00 D threshold (Table 2). However, reanalysing data using a ≥3.50 D threshold for hyperopia was still associated with a 0.05 logMAR cut-off point, limiting testing utility. Kleinstein et al. [14] defined emmetropia as less than 1.00 D hyperopia and 0.25 D myopia, removing ‘borderline’ cases between 1.00 and 2.00 D hyperopia, as well as between 0.25 and 0.75 D myopia, which may result in improved classification performance. When the present data were reanalysed using the definition of emmetropia reported by Kleinstein et al. [14], no notable changes in model performance were found (data not shown).

While a modest improvement in the classification of hyperopia cases was found when using near VA, performance remained poor. This is in contrast to a previous study reporting good AUC, with sensitivity and specificity values between 78% and 85%, when using near VA [16], likely due to including only children with hyperopia of ≥4.50 D SE. This may have improved their model performance. Similarly, while chance-level performance was found here for near VA in classifying myopia, the previous report found AUC, sensitivity and specificity values between 85% and 91% in participants with ≥3.00 D myopia [16]. Given the 2.50 D (40 cm) viewing demand for near VA testing, myopes in the present study (defined as ≥0.75 D myopia) were more likely to pass near acuity testing than those in the previous report [18].

Age-normed distance and near VA measures showed slightly weaker associations with general refractive error than unadjusted VA. Age-normed thresholds were used to account for lower attentional and cognitive capacity during VA testing in younger children [17, 18], but the current findings align with previous vision screening studies that reported age-normed thresholds to be ineffective in detecting underlying refractive error (see All RE in Table 3) [30]. This demonstrates that established vision screening guidelines based on VA may not accurately determine the need for a referral to an eye care provider for children whose reduced VA is due to refractive error [31]. In addition, the current linear regression findings demonstrated that age was not a significant predictor in determining distance VA levels within each refractive error classification. However, age significantly predicted near VA levels in myopia, whereby an improvement of 0.10 logMAR was observed for every additional 3 years of age in the sample of children aged 3 to <10 years. As mentioned, myopic participants in the current study did not experience notable defocus during near VA testing. Therefore, it is possible that VA may improve slightly with age when defocus is not a limiting factor.

Further, the current study found that the magnitude of myopic refractive error significantly predicted distance VA levels in children. A 0.10 logMAR decline was predicted for every additional 0.36 D of myopia, in line with previous reports [14, 32]. Coupled with the findings from the ROC curve analysis, this suggests that decreased distance VA is associated with myopia, with higher levels of myopia predictive of worse distance VA levels. Decreased VA was also associated with astigmatism; however, in contrast to previous studies [14, 32–34], the present data indicated no notable association between the magnitude of astigmatism and distance VA. The discrepancy in findings could be due to methodological differences. Most notably, the present sample only included 15 children with astigmatism-only, 12 (80%) of whom were aged ≤6 years. Previous studies that found an association between the magnitude of astigmatism and VA also examined a wider range of astigmatism levels [34], including cases of low or no astigmatism [33], which would impact the slope of the regression line. In a previous study, some individuals with astigmatism also presented with simultaneous myopia (or hyperopia) [33], which may influence the relationship between astigmatism and VA, whereas the current investigation only considered cases of astigmatism without concurrent classifications of myopia and hyperopia. It is also possible that the discrepancy in findings could be, in part, due to the current sample of children having no history of refractive correction, whereas previous studies assessed best-corrected VA in some or all children [33–35]. Investigations with adults have also shown long-lasting visual adaptation to an astigmatic refractive correction that impacts VA and blur perception; thus highlighting that short periods of refractive wear can influence VA [21]. Finally, distance VA was not only poorly associated with hyperopia, but higher magnitudes of hyperopic refractive error were also not associated with worse distance VA, consistent with an earlier study reporting no notable changes in VA in 6- to 14-year-olds with hyperopia and low levels of astigmatism [14].

Limitations of the current study include the smaller sample size of participants with clinically significant refractive error, which may limit the precision of estimates from the regression analyses. Given that broader refractive error categories were also required, more detailed categories (e.g., compound hyperopic astigmatism) could not be evaluated. Pupil size, which can affect depth of focus, as well as the astigmatic axis, was also not assessed and could potentially influence VA.

## Conclusion

This study demonstrates that despite its expected utility for identifying myopia and astigmatism, distance and near VA testing showed poor performance in classifying clinically significant hyperopia among 3- to <10-year-old children, even when age-normed acuity thresholds were applied. While higher magnitudes of myopia predicted worse distance VA, such associations were lacking for hyperopia and astigmatism. Thus, in clinical settings, distance VA can be used to corroborate the presence of myopic refractive error; however, eye care providers should not attempt to correlate reduced VA and the magnitude of astigmatic or hyperopic refractive error in young children.

## Data Availability

The data sets generated and analysed in this study are not publicly available due to patient privacy considerations.
